# Model Application of Entomopathogenic Fungi as Alternatives to Chemical Pesticides: Prospects, Challenges, and Insights for Next-Generation Sustainable Agriculture

**DOI:** 10.3389/fpls.2021.741804

**Published:** 2021-09-30

**Authors:** Bamisope Steve Bamisile, Komivi Senyo Akutse, Junaid Ali Siddiqui, Yijuan Xu

**Affiliations:** ^1^Department of Entomology, South China Agricultural University, Guangzhou, China; ^2^Plant Health Theme, International Centre of Insect Physiology and Ecology, Nairobi, Kenya

**Keywords:** biological control, plant-fungi interactions, integrated pest management, mutualism, plant nutrients, mycopesticides

## Abstract

In the past few decades, the control of pests and diseases of cultivated plants using natural and biological measures has drawn increasing attention in the quest to reduce the level of dependence on chemical products for agricultural production. The use of living organisms, predators, parasitoids, and microorganisms, such as viruses, bacteria, and fungi, has proven to be a viable and sustainable pest management technique. Among the aforementioned, fungi, most importantly the insect-pathogenic species, have been in use for more than 150years. These include the most popular strains belonging to the genera *Beauveria*, *Metarhizium*, *Isaria*, *Hirsutella*, and *Lecanicillium*. Their application is usually through an inundative approach, which inherently involves exposure of the fungal spores to unfavorable humidity, temperature, and solar radiation conditions. These abiotic factors reduce the persistence and efficacy of these insect-pathogenic fungi. Despite these limitations, over 170 strains have been formulated as mycopesticides and are available for commercial use. In the last few decades, numerous studies have suggested that these species of entomopathogenic fungi (EPF) offer far more benefits and have broader ecological functions than hitherto presumed. For instance, aside from their roles as insect killers, it has been well established that they also colonize various host plants and, hence, provide other benefits including plant pathogen antagonism and plant growth promotion and serve as sources of novel bioactive compounds and secondary metabolites, etc. In this light, the potential of EPF as alternatives or perhaps as supplements to chemical pesticides in plant protection is discussed in this review. The paper highlights the numerous benefits associated with endophytic fungal entomopathogen and host plant associations, the mechanisms involved in mediating plant defense against pests and pathogens, and the general limitations to the use of EPF in plant protection. A deeper understanding of these plant host-fungus-insect relationships could help unveil the hidden potentials of fungal endophytes, which would consequently increase the level of acceptance and adoption by users as an integral part of pest management programs and as a suitable alternative to chemical inputs toward sustainable crop production.

## Introduction

Insect pests, plant pathogens, and unfavorable growing conditions induce biotic and abiotic stresses in crop plants. These factors are responsible for huge plant productivity losses (up to 26% crop losses, valued at over $470 billion worldwide; Culliney, 2014). To ensure optimum productivity of cultivated plants, agriculturists depend heavily on chemical insecticides and inorganic fertilizers to combat these problems (Skinner et al., 2014). The overdependence on synthetic fertilizers for improving the growth of plants is another constraint faced by agriculturists. This is because the overreliance on these chemicals poses several side effects to users, non-target organisms, and the environment (Skinner et al., 2014; Fadiji and Babalola, 2020b).

As the population of the world is expected to increase to approximately 9.1 billion by 2050 (Liu et al., 2017a), efforts are in place to ensure sustainable agricultural production. However, excessive usage and overdependence on synthetic pesticides and fertilizers, climatic changes, poor land management, and mass urbanization are some of the factors affecting these efforts (Smith et al., 2016). The potential application of entomopathogenic fungi (EPF) as biocontrol agents against herbivores represents an environmentally sustainable alternative insect pest management approach (West and Gwinn, 1993). EPF are known for their ability to infect insects leading to disease in proper conditions, where they directly colonize the insect body by penetrating its cuticles. To date, more than 700 species from approximately 90 different genera have been established as insect-pathogenic fungi (Khachatourians and Qazi, 2008). These include the most popular strains belonging to the genera *Beauveria*, *Metarhizium*, *Isaria*, *Hirsutella*, and *Lecanicillium* (Inglis et al., 2001; Khachatourians and Qazi, 2008). Among them, *Beauveria bassiana* (Balsamo-Crivelli) Vuillemin, *Isaria fumosorosea* Wize, *Metarhizium anisopliae* (Metschnikoff) Sorokin, and *Lecanicillium lecanii* (Zimmerman) Viegas are the most commonly studied fungal species (Li et al., 2011; Chen et al., 2015). These entomopathogenic fungal strains are commonly studied for their potential use as biological control agents in mitigating crop losses due to insect pests (Hunter, 2005). *Beauveria bassiana* and *M. anisopliae* are the most widely distributed species and are commonly found on and have been isolated from infected insects in both temperate and tropical regions throughout the world (Zimmermann, 2007a). Several of the EPF species, for example, *I. fumosorosea* and *I. farinosa*, can infect multiple hosts without showing any of the numerous harmful effects associated with chemical pesticides and, therefore, are considered safe and environmentally friendly (Gao et al., 2017). These EPF, aside from naturally regulating insect populations by causing epizooties, have also been established to play additional multitrophic roles. They have the ability to colonize different host plants and exist in the form of fungal endophytes (Vega et al., 2009; Qayyum et al., 2015; Bamisile et al., 2018b; Wakil et al., 2020), act as rhizosphere colonizers (Hu and St Leger, 2002), serve as plant pathogen antagonists (Ownley et al., 2004, 2010; Kim et al., 2008; Jaber and Ownley, 2018), plant growth promoters/biofertilizers (Kabaluk and Ericsson, 2007; Elena et al., 2011; Sasan and Bidochka, 2012; Liao et al., 2014; Lopez and Sword, 2015; Jaber and Enkerli, 2017), and as sources of novel bioactive compounds and multiple secondary metabolites (Tadych et al., 2009; Hu et al., 2016; Al-Ani et al., 2021). In addition, they can also play an essential role in the biotransformation of steroids and flavonoid glycosides (Dymarska et al., 2017, 2018). Several studies have reported different insect-pathogenic fungal species as natural colonizers/endophytes of many economically important crops, including maize, coffee, potato, cotton, beans, Jimson weed, tomato, and chickpea (Jones, 1994; Arnold and Lewis, 2005; Vega et al., 2008; Qayyum et al., 2015; Wakil et al., 2020). Similarly, the potential for establishing these EPF strains as endophytes in different plant species using various artificial inoculation methods has also been previously discussed (Bamisile et al., 2018a; Sinno et al., 2020).

These numerous attributes of endophytic EPF ensure that, in addition to their conventional application as insect killers, they can also be adopted as beneficial plant growth-promoting microorganisms, and they have shown great potential thus far as biofertilizers. These endophytic fungal species are believed to serve as alternatives to systemic fertilizers, as well as an efficient and eco-friendly approach toward food security (Glick, 2014). In organic farming systems, the level of utilization of fungal endophytes as a means of improving yields and protecting plants from damage is increasing (Shrivastava et al., 2010). Various species of endophytic fungi have been underlined for their potential as indirect biocontrol agents in large-scale agricultural applications (Lacey and Neven, 2006). The use of biotechnology for crop improvement through inoculation of plants with modified fungal strains would therefore reduce toxicity to humans, livestock, and the environment. The genes of fungal endophytes could be genetically transformed through the removal of detrimental genes or otherwise by the addition of new beneficial genes (Adeleke and Babalola, 2021). Endophytes could then be used as surrogate hosts to transform crops genetically. Using this method, the endophyte of ryegrass has been genetically transformed and successfully applied as a deterrent to herbivores (Murray et al., 1992). Similarly, in the quest to improve endophytic resources, efforts are being made toward the discovery of novel metabolic compounds that cannot otherwise be synthesized using chemical methods. It is therefore imperative to have a clear understanding of the biology of plants and the ecology of the fungal communities to explore the richness of beneficial fungal endophytes under different cropping systems. In addition, to make fungal entomopathogens readily available and easy to use, as they are considered as biocontrol agents with a non-resistance and non-contaminant advantage over synthetic pesticides, many insect-pathogenic fungal strains have been formulated as bioinsecticides (Fang et al., 2014) and, thus, are currently considered an alternative management method for many insect pests of economic value. Due to the aforementioned attributes and many other prospects, the level of acceptance and adoption of fungal entomopathogens/endophytes is rapidly increasing, and thus, research into their biology, ecology, and mode of action is attracting more public and scientific interest (Dong et al., 2016; Hu et al., 2016). In this light, the current review discusses the available knowledge on EPF utilization and mechanisms as biological agents for plant growth promotion and pest and disease control, thereby exploring the prospects and limitations toward potential adoption as alternatives to synthetic pesticides.

## Entomopathogenic Fungi as Alternatives to Chemical Pesticides: A Reality or Myth?

Entomopathogenic fungi have been in active use for the management of a plethora of economic pests of crop plants for approximately 200years now. *Beauveria bassiana* was first isolated and identified approximately 170years ago (Zimmermann, 2007b), while *Beauveria brongniartii* (Saccardo) Petch and *M. anisopliae* have also been in use for over 110 and 130years, respectively (Zimmermann, 2007a). These fungal species together with other known hypocrealean fungi, such as *I. fumosorosea*, *M. brunneum*, *M. robertsii*, and *Hirsutella thompsonii* Fisher, are commonly used against a broad range of arthropod pests (Dara, 2019b). They are mostly applied through inundative approaches (Bamisile et al., 2018b; Jaber and Araj, 2018) and have been reported to be effective against several insects of different feeding guilds including aphids, locusts, thrips (Gulzar et al., 2021), grubs (Wakil et al., 2017; Yasin et al., 2019), moths (Ali et al., 2015; Tahir et al., 2019), mites, mosquitoes, whiteflies, and tephritid fruit flies (Dong et al., 2016; Bamisile et al., 2020; Canassa et al., 2020; Usman et al., 2020). Additionally, EPF have been found to be pathogenic against phytopathogenic nematodes and other soil-borne pests (Pocasangre et al., 2000).

The management of economic pests using insect-pathogenic fungi, therefore, serves as an effective and sustainable alternative to chemical control. Despite the enormous benefits of EPF, the exposure of fungal spores to unfavorable climatic conditions in the field reduces their efficiency and level of general utilization (Dong et al., 2016). However, utilization of EPF through inoculation as fungal endophytes rather than using an inundative approach can help significantly reduce the negative effects due to abiotic stressors (Vega, 2018). For instance, it has been reported that *B. bassiana* can offer longer protection to the host plant when existing as an endophyte *in planta*. The fungus can persist in the host tissues over a long duration. This possibility has been reported in citrus (Bamisile et al., 2020), jute, *Corchorus olitorius* (Biswas et al., 2012), and radiata pine, *Pinus radiata* (Brownbridge et al., 2012), where endophytic colonization of the hosts was found to be sustained up to 2, 3, and 9 months post-fungal treatment of seedlings, respectively. Several studies also reported similar effects and properties in different plant species, including coffee, fava bean, and common bean (Posada et al., 2007; Jaber and Enkerli, 2016; Dash et al., 2018). In this regard, there are significant pieces of evidence to confirm that EPF can be successfully introduced as fungal endophytes in plants using different artificial inoculation methods (Bamisile et al., 2018a) and could consequently be used as substitutes for chemical pesticides. In addition, some previous studies have reported systemic colonization of treated plants without any symptomatic effects. This implies that treatment of a specific organ or part of the plant (leaf, stem, or root) irrespective of the artificial inoculation method used can result in endophytic colonization of the entire plant and confer systemic resistance to the host plant against herbivores (Mantzoukas et al., 2015; Jaber and Enkerli, 2016; Dash et al., 2018).

Successful endophytic colonization of plants provides multiple benefits, including plant growth promotion, protection against insect pests, induction of systemic resistance, antagonization of plant pathogenic fungi, bacteria, and nematodes, and suppression of the negative effects of abiotic stressors on host plants (Kabaluk and Ericsson, 2007; Kim et al., 2008; Vega et al., 2009; Ownley et al., 2010; Elena et al., 2011; Sasan and Bidochka, 2012; Liao et al., 2014; Vega, 2018). In addition to the aforementioned benefits of EPF/endophytes, in the past few decades, fungal endophytes have garnered more attention, as well as broader biotechnological and industrial relevance, due to their uniqueness as sources of secondary metabolites. Their ability to secrete novel biochemical compounds arguably provides an edge over chemical pesticides. Furthermore, they serve medical/pharmaceutical purposes as antimicrobial, antidiabetic, antitumor, and immune suppressing agents (Gouda et al., 2016; Yadav, 2018). This aspect is discussed further in the section “*endophytic fungi as good sources of pharmaceutical products*.” As a rich source of natural products, it is worth noting that in the last 20years, these organisms have been isolated from various plants for industrial and agricultural purposes (Fadiji and Babalola, 2020a).

Many studies have underlined the important roles played by endophytic fungi, and their non-pesticidal properties are of huge importance in fungal evolution and survival in plants and in soil environments in the absence of arthropod hosts (Dara, 2019b). Many studies have provided evidence that confirms that these beneficial microbes can improve the soil structure and microbiome, and nutrient and water absorption in plants, induce systemic resistance, and serve as probiotics that antagonize harmful microorganisms (Sasan and Bidochka, 2012; Jaber and Salem, 2014; Behie et al., 2015; Jaber and Alananbeh, 2018). Another interesting aspect of the use of endophytic fungi as insect pest biocontrol agents is that some of the notorious pests that have developed resistance or otherwise successfully evaded chemical pesticide treatment have been shown to be successfully controlled using these EPF/endophytes. Examples of such pests are stem borers, which have been reported to be susceptible following treatment of coffee (Posada and Vega, 2006) and sorghum plants (Tefera and Vidal, 2009) with endophytic *B. bassiana*.

In recent years, with the increasing interest in sustainable food production systems, biological agents, including beneficial microbes, biostimulants, and other biocontrol agents, have been adopted and are now gaining more popularity. Considering the unique attributes of endophytic fungi, most of these species are currently being explored for crop production in both small- and large-scale farming and in home and community gardens (Dara, 2019b). With reference to the aforementioned benefits and many more that are still emerging and the numerous prospects of endophytic EPF, the possibility of becoming a suitable replacement for inorganic chemicals is rapidly becoming a reality.

## Formulation of Entomopathogenic Fungi as Mycopesticides

Multiple genera of hypocrealean fungi that have been found effective against various species of arthropod pests are considered an integral component of integrated pest management (IPM) strategies in maintaining pest control efficacy, mitigating the risk of inorganic pesticide resistance, and offering environmentally sustainable pest suppression (Dara, 2019b). To achieve these objectives, many fungal entomopathogen-based biopesticides have been formulated over the years, as they are believed to be suitable and direct replacements of the commonly used synthetic insecticides. Although the history of mycoinsecticide and mycoacaricide development dates back to the early 1960s (de Faria and Wraight, 2007), it is of note that the frequencies of applications and timings of most of these mycoinsecticides are similar to those of conventional insecticides (Wraight et al., 2000; Shah and Pell, 2003). The most common mycopesticides are products formulated from *B. bassiana*, *M. anisopliae*, *B. brongniartii*, and *I. fumosorosea*. For instance, in the last three decades, a good number of *M. anisopliae-* and *B. bassiana*-based mycoinsecticides have been commercialized and registered in various countries (Zimmermann, 2007a,b; more detail is provided in Table 1; see also Wraight et al., 2001; de Faria and Wraight, 2007).

**Table 1:** Some of the common mycopesticides that have been formulated and registered for use as alternatives to chemical pesticides.

Source	Trade name of product	Name of manufacturing company	Country of origin	Target insect pests	Reference
*B. bassiana*	Bauveril	Laverlam S.A., Colombia	Colombia, Dominican Republic	Coleoptera (Curculionidae, Scarabaeidae), Lepidoptera (Castniidae)	de Faria and Wraight, 2007
	Bio-Power	Stanes	India	Stem borers, cut worms, root grubs, leafhoppers, whiteflies, aphids, thrips and mealy bugs.	de Faria and Wraight, 2007; Zimmermann, 2007a
	BotaniGard ES BotaniGard 22 WP	Laverlam International (formerly Emerald BioAgriculture)	United States	Grasshoppers, whiteflies, thrips, aphids and many other insect pests	Shah and Pell, 2003; de Faria and Wraight, 2007
	Boverol	Fytovita	Czech Republic	Coleoptera (Chrysomelidae)	de Faria and Wraight, 2007; Zimmermann, 2007a
	Boverin	Biodron	Russia	Colorado potato beetle; *Leptinotarsa decemlineata*, and the codling moth; *Cydia pomonella*	Zimmermann, 2007a
	Conidia	Hoechst Schering AgrEvo	Columbia	Coleoptera (Curculionidae)	de Faria and Wraight, 2007; Zimmermann, 2007a
	Mycotrol ES Mycotrol-O	Laverlam International (formerly Emerald BioAgriculture)	United States	Grasshoppers, whiteflies, thrips, aphids, and many other insect pests	Shah and Pell, 2003
	Naturalis	Intrachem	Italy	Coleoptera (Chrysomelidae, Curculionidae, Scarabaeidae), Hymenoptera (Formicidae), Diptera (Tipulidae), Hemiptera (Lygaeidae, Cercopidae, Cicadellidae, Aleyrodidae, Aphididae, Pseudococcidae, Psyllidae)	Zimmermann, 2007a
	Naturalis-L	Andermatt Biocontrol Troy Biosciences Inc.	Switzerland United States	Coleoptera (Chrysomelidae, Curculionidae), Hemiptera (Miridae, Cicadellidae, Aleyrodidae, Aphididae, Psyllidae), Lepidoptera, Thysanoptera (Thripidae)	de Faria and Wraight, 2007; Zimmermann, 2007a
	No tradename	Anhui Heibaogong Pest Control Co., Ltd	China	Unknown	Website^1^
	No tradename	Shandong Ruyi Biotechnology Co., Ltd	China	*Plutella xylostella* (Linnaeus)	Website^1^
	No tradename	Hebei Zhongbao green crop Technology Co., Ltd	China	Thrips	Website^1^
	Ostrinil	Arysta (formerly NPP, Calliope)	France	Lepidoptera (Crambidae)	Wraight et al., 2001; de Faria and Wraight, 2007; Zimmermann, 2007a
	Proecol	Probioagro S.A., Venezuela	Venezuela	Lepidoptera (Noctuidae)	Wraight et al., 2001; de Faria and Wraight, 2007; Zimmermann, 2007a
	Racer BB	SOM Phytopharma	India	Lepidoptera (Noctuidae)	de Faria and Wraight, 2007; Zimmermann, 2007a
	Trichobass-L Trichobass-P	AMC Chemical/Trichodex	Spain	Coleoptera (Curculionidae, Scarabaeidae), Lepidoptera (Castniidae, Pieridae), Hemiptera (Aleyrodidae), Thysanoptera (Thripidae)	de Faria and Wraight, 2007; Zimmermann, 2007a
*B. brongniartii*	Beauveria Schweizer	Lbu (formerly Eric Schweizer Seeds)	Switzerland	European cockchafer and other scarab beetles species	Shah and Pell, 2003
	Betel	Arysta (formerly NPP, Calliope)	France	Coleoptera (Scarabaeidae)	Zimmermann, 2007a
	Biolisa-Kamikiri	Nitto Denko	Japan	Coleoptera (Cerambycidae)	Wraight et al., 2001; de Faria and Wraight, 2007; Zimmermann, 2007a
	Engerlingspilz	Andermatt Biocontrol AG	Switzerland	European cockchafer and other scarab beetles species	Shah and Pell, 2003
	Melocont-Pilzgerste	Agrifutur-Kwizda	Italy Austria		Zimmermann, 2007a
*I. farinosa*	Paecilomin		Russia	Apple moth, Siberian pine caterpillar, and larch caterpillar	Weng et al., 2019
*I. fumosorosea*	NoFly^™^ WP	Natural Industries Inc.	United States	Whiteflies, aphids, thrips, psyllids, mealybugs, and fungus gnats	Weng et al., 2019
*L. lecanii*	Mycotal	Koppert Biological Systems	Netherlands	Whiteflies and thrips	Shah and Pell, 2003
	Vertalec	Koppert Biological Systems	Netherlands	Aphids	Shah and Pell, 2003
*M. anisopliae*	Achieve	Real IPM	Mozambique	*Tetranynchus urticae*	Akutse et al., 2020
	BioBlast	EcoScience	United States	Isoptera (Kalotermitidae, Rhinotermitidae, Termopsidae)	de Faria and Wraight, 2007; Zimmermann, 2007b
	Bio-Cane	Granules Becker-Underwood	Australia	Sugarcane pest; grayback canegrub	Zimmermann, 2007b, Website^2^
	Bio-Catch-M	Stanes	India	Hemiptera (Aleyrodidae, Aphididae)	de Faria and Wraight, 2007; Zimmermann, 2007b
	Bio-Green Granules	Becker-Underwood	Australia	Coleoptera (Scarabaeidae)	de Faria and Wraight, 2007; Zimmermann, 2007b
	Bio-Magic	Stanes	India	Coleoptera (Curculionidae, Scarabaeidae), Hemiptera (Cercopidae)	de Faria and Wraight, 2007; Zimmermann, 2007b
	BioPath	EcoScience	United States	Blattodea (Blattellidae, Blattidae)	Zimmermann, 2007b
	Campaign	Real IPM	Ghana	Mealybugs	Akutse et al., 2020
			Uganda	Thrips, fruit flies, mealybugs	
	Cobican	Probioagro	Venezuela	Coleoptera (Scarabaeidae), Hemiptera (Cercopidae, Aphididae)	de Faria and Wraight, 2007; Zimmermann, 2007b
	Gran Met-P	Kwizda/Agrifutur	Austria/Italy	Coleoptera (Scarabaeidae, Curculionidae, Nitidulidae)	de Faria and Wraight, 2007; Zimmermann, 2007b
	Green Guard SC	Becker-Underwood	Australia	Orthoptera (Acrididae)	Zimmermann, 2007b
	Green Guard ULV	Becker-Underwood	Australia	Orthoptera (Acrididae)	Zimmermann, 2007b
	Green Muscle	CABI Bioscience/NPP	United Kingdom/France	Locust and grasshopper	Shah and Pell, 2003
	Mazao achieve	Real IPM	Kenya	*T. urticae*	Akutse et al., 2020
	Mazao campaign	Real IPM	Kenya	Mealybugs	Akutse et al., 2020
	Mazao supreme	Real IPM	South Africa	Aphids	Akutse et al., 2020
	Metaquino		Brazil	Sugarcane spittle bug; *Mahanarva postica*	Zimmermann, 2007b
	Metarhizium Schweizer	Lbu (formerly Eric Schweizer Seeds)	Switzerland	Coleoptera (Scarabaeidae)	Zimmermann, 2007b
	Metathripol	ICIPE	Kenya	Thrips	Website^3^
	No tradename	Chongqing Zhongda Biotechnology Development Co., China	China	Orthoptera (Acrididae)	Nong et al., 2015
	No tradename	Jiangxi Tianren Ecology Corp., China	China	Lepidoptera (Noctuidae)	Nong et al., 2015
	No tradename	Beijing Sangbai Biotechnology Co., Ltd., China	China	Blattaria	Nong et al., 2015
	Pacer	SOM Phytopharma	India	Isoptera	Zimmermann, 2007b
	Real metarhizium 69	Real IPM	South Africa	Mealybugs, thrips, leafminers	Akutse et al., 2020
			Zambia	Fruit flies, mealybugs	
	Real metarhizium 78	Real IPM	Canada	*T. urticae*, plant growth regulator	Akutse et al., 2020
	Real metarhizium OD	Real IPM	Zimbabwe	Biofertilizer	Akutse et al., 2020
	Real metarhizium SC	Real IPM	Tanzania	Mealybugs	Akutse et al., 2020
	Taenure Granular Bioinsecticide	Novozymes Biologicals (formerly Earth BioSciences)	United States	Coleoptera (Curculionidae, Scarabaeidae), Diptera (Ephydridae, Mycetophilidae, Sciaridae, Tipulidae), Thysanoptera (Thripidae)	Zimmermann, 2007b, Website^4^
	TAE-001 Technical Bioinsecticide	Novozymes Biologicals (formerly Earth BioSciences)	United States	Coleopterans; Elateridae, Curculionidae	Zimmermann, 2007b
	Tick-Ex EC	Novozymes Biologicals (formerly Earth BioSciences)	United States	Acari (Ixodidae), Coleoptera (Scarabaeidae)	Zimmermann, 2007b
	Tick-Ex G	Novozymes Biologicals (formerly Earth BioSciences)	United States	Acari (Ixodidae), Coleoptera (Scarabaeidae)	Zimmermann, 2007b

1 *Website: http://www.chinapesticide.org.cn/hysj/index.jhtml (In Chinese)*.

2 *Website: https://www.biosciregister.com/Becker_Underwood/Supplier/sid2909.htm*

3 *Website: https://sitem.herts.ac.uk/aeru/bpdb/Reports/1980.htm*

4 *Website: http://www.epa.gov/oppbppd1/biopesticides/ingredients/*

The spores of *B. bassiana* and *B. brongniartii* have been successfully formulated as mycopesticides in many countries, and several of these products have passed the registration requirements and are therefore currently widely used for biocontrol of pest insects in various countries (Wraight et al., 2001; de Faria and Wraight, 2007). *Metarhizium anisopliae* strains have also been developed and commercialized against several pests and many disease vectors (Akutse et al., 2020). Similarly, *L. lecanii* has been reported to naturally control aphid and scale insect populations in tropical and subtropical regions and thus has been studied and formulated for use as a mycoinsecticide. According to Shah and Pell (2003), *L. lecanii* was the first fungus to be developed as inundative mycoinsecticide for use in medium- to large-scale farming in glasshouses. The active ingredients from two different isolates were formulated into two products: “Vertalec” against aphids and “Mycotal” for the control of whiteflies and thrips (Shah and Pell, 2003). Both products have been registered in numerous countries in Europe and beyond, including the Netherlands, Finland, Denmark, France, Norway, Turkey, Spain, and the United Kingdom. Since their introduction in 1981, strong efficacy against a broad range of aphid species has been reported (Yeo et al., 2003). Another popular product that has been developed for use is “Mycotrol,” which is a mycoinsecticide formulated from *B. bassiana* (Bradley et al., 1992). The product was registered in 1999 for use against aphids, grasshoppers, thrips, whiteflies, and many other insect pests affecting trees and field crops. Similarly, another mycopesticides, “Green Muscle,” was developed in Africa to control the outbreak of the desert locust, *Schistocerca gregaria* Forskal (Orthoptera: Arcidridae), between 1985 and 1989. The research project was undertaken by research institutions in the United Kingdom, the Netherlands, the Republic of Benin, and Niger. The mycoinsecticide was made of dried conidia of *Metarhizium anisopliae* var. *acridum*, which is often mixed with kerosene or diesel oil before application (Bateman et al., 1998). The product was reported to cause up to 90% mortality in treated grasshoppers and locusts within 2–3weeks post-treatment, while no side effects on non-target organisms were recorded (Lomer et al., 2001). Before this period, around the early 1980s in Russia (then USSR), a *B. bassiana*-based mycopesticide named “Boverin” was applied over thousands of hectares for the control of the Colorado potato beetle, *Leptinotarsa decemlineata* Say (Coleoptera: Chrysomelidae), and the codling moth, *Cydia pomonella* Linnaeus (Lepidoptera: Tortricidae; Hussey and Tinsley, 1981). Several other similar products are available for use in glasshouses, as well as by organic farmers in the United States and beyond. Examples of such formulations are “BotaniGard” and “Mycotrol-O.” All of these products are considered suitable replacements for synthetic insecticides.

## Benefits and Safety Concerns of Biopesticides

In recent years, the numerous benefits that could be derived from mycopesticide utilization in place of chemical insecticides have been highlighted by many authors (Wraight et al., 2001; Shah and Pell, 2003; de Faria and Wraight, 2007; Zimmermann, 2007a,b). More advanced scientific investigations on how to manage the various setbacks encountered and improve the efficiency of these microbial organisms are still emerging. However, the most common questions raised, which have been attracting the attention of several researchers and mycopesticide users, are concerns about the safety risks of biopesticides and their secondary metabolites. Zimmermann (2007a) analyzed the safety issues related to *Beauveria* spp. use as mycopesticides, where the author highlighted the biological properties, history, geographical distribution, host range, mode of action, and toxin-producing capabilities of the fungal species. The potential side effects on non-target organisms, such as predators, parasitoids, pollinators, arthropods, and vertebrates (birds, fish, amphibians, and reptiles), and human health were also discussed. The author suggested that, to date, no serious side effects have been ascribed to the use of the two *Beauveria* strains and hence concluded that both *B. bassiana* and *B. brongniartii* are relatively safe for use as mycopesticides (Zimmermann, 2007a). Other recent studies have also confirmed the low-risk status of common mycoinsecticides and therefore proposed their use as alternatives to chemical insecticides for the management of agricultural pests and disease vectors (Zimmermann, 2007a,b). The safety of these products to users and the environment has been well-assessed (Haas-Costa et al., 2010). In addition, the mycotoxins produced by them are considered very unlikely to enter the food chain (Hu et al., 2016).

## Entomopathogenic Fungi Mediating Plant Defense Against Insect Pests

Many species of insect-pathogenic fungi have been characterized for their ability to colonize and become established as fungal endophytes in plants. *Beauveria bassiana*, *M. anisopliae*, and other hypocrealean fungi are known to colonize many plants endophytically (Vega et al., 2008). Several studies have demonstrated the colonization potential of various EPF using different artificial inoculation techniques. Endophytic fungi can be inoculated into plants using foliar application, soil drench, flower treatment, stem injection, seed soaking, etc. (Lopez and Sword, 2015; Muvea et al., 2015; Greenfield et al., 2016; Bamisile et al., 2018b; Rondot and Reineke, 2018; Ramos et al., 2020). The inoculation method may depend on the part of the plant targeted for endophytic colonization or the type of insects to be controlled, that is, root eater, stem borer, or leaf chewing insect (Bamisile et al., 2018a). However, irrespective of the inoculation method used, many of the recent studies have revealed several tritrophic interactions that exist among the inoculated plants, the endophytic EPF, and the herbivores feeding on the endophyte-challenged plants (Akello et al., 2008; Vega et al., 2008; Reddy et al., 2009; Dash et al., 2018).

Fungal endophytes generally live part or their lifecycle within the tissues of living hosts without causing any noticeable disease symptoms (Suryanarayanan, 2013; Hardoim et al., 2015). They can colonize any part of the host, including the embryo of seeds. As the seedlings germinate and develop during the early growth stages, the endophytes also increase in abundance (Shade et al., 2017). Endophytes depend on their plant hosts for nutrition and provide indirect defense against herbivores associated with the hosts (Backman and Sikora, 2008; Tadych et al., 2009). The endophytic fungi involved in this fungus-plant interaction have been described as plant-defending mutualists (Saikkonen et al., 2004), where the fungi mediate adaptive protection against insect pests of the host plant (White et al., 2002). Several recent studies have demonstrated that endophyte-challenged crop plants are less likely to be attacked by insect pests (Lopez and Sword, 2015; Muvea et al., 2015; Rondot and Reineke, 2018; Ramos et al., 2020). In addition, evidence of endophytic fungi reducing the productivity of herbivores feeding on colonized plants is readily available (Vega et al., 2009; Lopez et al., 2014; Dash et al., 2018; Dara, 2019b). Fungal endophytes are believed to serve as bodyguards for their host plants against primary herbivore pests (Vidal and Jaber, 2015; Jaber and Ownley, 2018). Under moderate to high levels of herbivore attack, plants colonized with endophytic fungi can generally outperform endophyte-free plants (Clay, 1997).

## Mode of Action Against Insect Pests

When present in the form of fungal endophytes in plants, EPF induce indirect detrimental effects on pests through various non-pathogenic mechanisms, such as antibiosis, antixenosis, and induced systemic resistance (ISR; Hartley and Gange, 2009). In this case, the fungus provides indirect defense against the host pests in exchange for carbohydrate energy resources derived from the plant (Wang and Qiu, 2006). The fungal endophytes, which provide indirect defense against their hosts’ primary enemies, may have been derived from different origins, including mutualistic root endophyte associations and the evolution of EPF into plant-associated endophytes (Vega et al., 2008). This plant-fungus mutualist interaction has been found to increase the rate of water and nutrient absorption as well as providing protection from insect pests, birds, and mammals (Lekberg and Koide, 2005). The mechanisms by which endophytic fungi minimize insect herbivore damage in their host plants are numerous, including pest avoidance or deterrence (Latch et al., 1985), reduction in feeding (Knoch et al., 1993), survival (Lacey and Neven, 2006), oviposition (Clay, 1990), and growth and developmental rate (Valenzuela-Soto et al., 2010).

As new findings are emerging periodically, various scientific studies are ongoing, and many others are still to come. Several questions have been raised, and different authors have identified various research gaps in very recent publications. In this light, the mechanisms underlying the pathogenicity-related activities of endophytic fungi against insect pests might not have been fully explored (Vidal and Jaber, 2015; Vega, 2018). In general, endophytic fungi promote host protection against primary pests by stimulating the production of plant defensive compounds, which have been characterized as having numerous bioactivities and functions (Carroll, 1988). Fungal endophyte-challenged plants exhibit feeding deterrence or antibiosis against their primary insect pests due to the synthesis of secondary metabolites by endophytic fungi. Colonized plants are less favorable to herbivores and indirectly affect the fecundity, fitness, and longevity of pests (Akello and Sikora, 2012; Akutse et al., 2013; Muvea et al., 2014; Mantzoukas et al., 2015; Dash et al., 2018; Jaber and Ownley, 2018). Endophytic fungi belonging to the genera *Beauveria* and *Metarhizium* spp. are commonly known for their ability to synthesize different arrays of secondary metabolites, which have been reported to exhibit antibacterial, antifungal, and insecticidal properties. These compounds include bassianolides, bassianolone, beauvericin, and oosporein, which are synthesized by *B. bassiana*. Similarly, cytochalasins, destruxins, serinocyclins, etc., are key compounds derived from *Metarhizium* spp. (Krasnoff et al., 2007). For instance, a hexa-cyclodepsipeptidic mycotoxin known as destruxin A (DA), synthesized by *M. anisopliae*, has been revealed to exhibit insecticidal and immunosuppressing activities (Fan et al., 2013; Ravindran et al., 2016).

Following endophytic colonization of plants, the fungus alters the nutrient content of the host to favor the production of secondary metabolites. Alterations in the chemical composition of the host plant inhibit the rate of herbivory and oviposition by insects (Clay, 1990). The detrimental effects of endophytic fungi and their metabolites on insect pests are different from those of the fungal infections caused by herbivore exposure to conidia or blastospores. The fungus grows as mycelia inside the plant, while infective structures are not produced inside the plant tissues as opposed to the hemolymph of the infected host insect. As a result, mycosis does not generally occur due to herbivores feeding on colonized plant tissues (Qayyum et al., 2015). However, there are a few records of mycosis in insect cadavers (Powell et al., 2009; Ramakuwela et al., 2020). The most common examples are in chewing insects, where insects feeding on colonized plants can easily be exposed to EPF emerging from wounded plant tissues. Following exposure, conidiation and infection could occur epiphytically, resulting in the dead insect showing mycosis. Powell et al. (2009) opined mycosis could have resulted from the exposed insect consuming an intact and sufficient amount of hyphae. There are a few available reports of mycosis in pests that fed upon host plants endophytically colonized by *B. bassiana*, such as Akello et al. (2008), Vidal and Jaber (2015), Klieber and Reineke (2016), and Ramakuwela et al. (2020). The negative effects of defense chemicals induced by fungal endophytes are more evident in generalist pests than in specialist pests. This is because generalists are more susceptible to endophytic fungal-mediated specific and qualitative defenses (Smith and Read, 2010). Koricheva et al. (2009) suggested that the negative effects of fungal infection on generalist pests could indirectly benefit specialist chewing insects. A similar finding has also revealed significant detrimental effects on generalist mesophyll feeders, while in contrast, phloem feeders were found to be less susceptible to fungal defense (Gehring and Bennett, 2009).

## Fungal Endophyte-Pathogen Interactions Mediating Host Resistance Against Plant Pathogens and Diseases

Endophytic fungi protect their host plants against pathogens by engaging similar mechanisms as those used in inducing plant resistance against herbivores (Jaber and Ownley, 2018). The secondary metabolites produced by these wide ranges of fungal endophytes have been found to exhibit antifungal and antibacterial potential, which help host plants evade damage/disease caused by phytopathogenic microorganisms (Gunatilaka, 2006).

In general, endophytic fungi mediate plant disease antagonism by inducing systemic plant resistance. The endophytes of the upper parts of grasses and some other beneficial plants have been well characterized for these activities (Jaber and Enkerli, 2016; Dash et al., 2018). Many species of endophytic EPF have been reported for their antibiotic and herbicidal properties. In addition, studies have revealed that these endophytic fungi may also influence plant pathogen associations by reducing their diversity and abundance (Tadych et al., 2009). Most importantly, for *B. bassiana* and *L. lecanii*, in addition to their well-known biological control activities against insect pests, both fungi have been revealed to possess antimicrobial and plant pathogen antagonism potential (Ownley et al., 2010; Jaber, 2018; Jaber and Ownley, 2018). For instance, the ability of *B. bassiana* to antagonize plant disease-causing pathogens in tomato, squash, cotton, grapevine, and many other economic crops has been reported (Ownley et al., 2004, 2008, 2010; Vega et al., 2009; Jaber and Ownley, 2018; Vega, 2018). The available reports have provided evidence of *Beauveria* sp. inhibiting various plant pathogens, including *Fusarium oxysporum*, *Botrytis cinerea*, *Septoria* sp., *Gaeumannomyces graminis*, *Pythium* sp., and *Rhizoctonia solani* (Renwick et al., 1991; Flori and Roberti, 1993; Veselý and Koubova, 1994; Bark et al., 1996; Lee et al., 1999). The level of infection by Zucchini yellow mosaic virus in squash was reduced following treatment of seedlings with *B. bassiana* (Jaber and Salem, 2014). Ownley et al. (2004) also reported a reduction in the severity of damping-off disease caused by *R. solani* in tomato. Aside from reports on *B. bassiana* and *L. lecanii*, other insect-pathogenic fungal strains have also been found to exhibit antagonistic properties against various arrays of phytopathogenic organisms. For example, *M. anisopliae* was found to minimize the spread of Dutch elm disease (DED), a vascular wilt disease caused by the ascomycete fungus, *Ophiostoma ulmi* Buisman (Gemma et al., 1984). Additionally, *M. brunneum* reduced the activities of *Fusarium culmorum* Smith – the causal agent of CRR in wheat (Jaber, 2018) and sweet pepper (Jaber and Alananbeh, 2018). In addition to the aforementioned reports, there are many other pieces of evidence of EPF interactions with phytopathogenic fungi (Kim et al., 2008; Sasan and Bidochka, 2012; Jaber, 2015; Jaber and Araj, 2018; Barra-Bucarei et al., 2020; Canassa et al., 2020).

## Mechanisms of Fungal Endophyte-Induced Plant Defense Against Pathogens

The mechanisms involved in fungal endophyte-induced plant defense against plant pathogens may not have been fully elucidated (Ganley et al., 2008); however, numerous modes of action through which this plant-endophyte mutualism helps hosts build resistance against pathogens have been proposed (Ownley et al., 2008, 2010; Gao et al., 2010; Jaber, 2018; Jaber and Ownley, 2018). The utilization of metabolites, which is the most popular and widely discussed indirect plant disease management strategy employed by the endophytic EPF in their hosts, is highlighted many times in this paper. The secretion of these unique biochemical compounds by endophytes helps to inhibit the evasion of harmful foreign microbes (Kusari et al., 2012). Some of the important metabolites, such as alkaloids, flavonoids, peptides, phenols, polyketides, quinones, steroids, and terpenoids, have been discovered from fungal endophytes and characterized in terms of their antimicrobial activities (Mousa and Raizada, 2013; Lugtenberg et al., 2016). The ability of these bioactive compounds to inhibit phytopathogens has been exclusively explored, as a good number of previous studies have identified novel metabolites from fungal endophytes that are suitable for commercial purposes (Suryanarayanan, 2013). Ongoing and future studies should focus on similar directions to explore the potential of these endophytes in phytopathogen and disease management. Fungal endophytes are unique for their ability to colonize the internal tissues of plants, an advantage they hold over many other biocontrol agents. In addition, the promotion of plant growth and initiation of systemic plant resistance are some of the other identified possible indirect mechanisms engaged by endophytic fungi. Several previous studies have established the possibility of fungal endophytes mediating systemic plant resistance and growth promotion in their hosts and hence reducing the damage caused by phytopathogenic microorganisms (Ganley et al., 2008). A systemic resistance strategy was observed in *B. bassiana*-treated pumpkin and cotton plants against zucchini yellow mosaic virus (Jaber and Salem, 2014) and *Xanthomonas axonopodis* pv. *malvacearum* (Ownley et al., 2008), respectively.

Generally, in response to attacks from parasites, pathogens, and other biotic and abiotic stressors, two different kinds of induced resistance patterns can be mediated by plants, namely, systemic acquired resistance (SAR) and ISR (Choudhary et al., 2007). SAR is activated upon exposure of the host to virulent or avirulent pathogens and other non-pathogenic microbes. This pathway is enhanced by the accumulation of the plant hormone, salicylic acid, and other pathogenesis-related proteins in the plant. Salicylic acid activates the SAR genes and prepares the plants for impending attack by a variety of pathogens in a quick and effective manner. The other pathway, ISR, is activated by the jasmonic acid and ethylene pathways following the activities of non-pathogenic microbes. Jasmonic acid production and pathogenesis-related protein activation are closely related to wounding in plants. Largely, the production of oxidative enzymes, such as polyphenol oxidases, peroxidases, and lipoxygenases, is involved in the latter pathway, while the production of antifungal pathogenesis-related proteins, including chitinases, 1, 3-glucanases, and thaumatins, is involved in the salicylate-induced pathway. Enzymes are directly involved in lysing foreign cells, cell wall strengthening, and cell death (Gao et al., 2010; Dara, 2019b).

Entomopathogenic fungal endophytes activate the production of plant defense proteins in their colonized host. This implies that the induced systemic responses produced by fungal endophytes are related to the enhancement of genes that are expressed in pathogenesis (Fadiji and Babalola, 2020a). Their ability to increase the production of pathogenesis-related proteins and other defense enzymes has been demonstrated by Karthiba et al. (2010) in rice and Senthilraja et al. (2013) in peanut, following dual treatment of both plants with *B. bassiana* and *Pseudomonas fluorescens*. The significant growth improvement recorded in the colonized plants was noticeable in plants resistance to the pathogen as well as an increase in the overall accumulation of peroxidase and polyphenol oxidase in the rice plants. Similar significant increases in the levels of catalase, chitinases, lipoxygenase, glucanase, phenolics, peroxidase, polyphenol oxidase, superoxide dismutase, and phenylalanine ammonia-lyase accumulated were recorded in the treated peanut seedlings. Similar findings were reported for date palm leaves following inoculation with *B. bassiana* and *Lecanicillium dimorphum* (Cordycipitaceae, Hypocreales; Gómez-Vidal et al., 2009). Furthermore, a strain of *B. bassiana* modified the gene expression levels across the phytoalexin, pathogenesis, salicylic acid, and jasmonic acid signaling pathways in *Arabidopsis*. As a result, the rate of *Sclerotinia sclerotiorum* (Libert) de Bary infection was significantly reduced in colonized *Arabidopsis* plants (Raad et al., 2019). The regulation of photosynthesis and energy metabolism-related proteins has also been reported. On the other hand, disease suppression *via* mycoparasitism, competition, and antibiosis have all been identified as some of the direct strategies employed by fungal endophytes against phytopathogens (Ownley et al., 2010; Jaber, 2018; Jaber and Ownley, 2018). For instance, while conducting both *in vitro* and *in vivo* observations of *Lecanicillium* spp. activities against *Pythium ultimum*, a ubiquitous soil-borne pathogen that causes damping-off and root rot infections in various plants, evidence of mycoparasitism between the two microorganisms was reported by Benhamou and Brodeur (2001). In another related study, Ownley et al. (2008) also found and reported a similar interaction between *Pythium myriotylum* and *B. bassiana*.

The ability of fungal endophytes to compete with disease-causing pathogens is another unique direct mechanism used to inhibit the colonization of their hosts by foreign microbes (Martinuz et al., 2013). Endophytic fungi are known to colonize their host, thereby hoarding the available nutrients and space and in turn limiting the activities of pathogens (Rodriguez et al., 2009). For instance, the ability of *B. bassiana* and *M. brunneum* to exhibit competition and antibiosis against *F. culmorum* upon subculturing both fungi in a dual plate assay with the fungal plant pathogen was revealed (Jaber and Alananbeh, 2018). Clear zones of inhibition across the interphase with the phytopathogenic fungus were observed, which provided evidence of competition for the available resources. The suppression or total removal of the endophytic fungi colonizing a plant through the application of fungicides, for instance, would allow the invasion of the plant tissues by other foreign microbes, as demonstrated in the study conducted by Mohandoss and Suryanarayanan (2009).

The mechanisms of competition utilized by fungal endophytes involve systematic colonization of parts of the host where foreign microbes could potentially colonize and, as a result, prevent further attack by the pathogen. In addition, fungal endophytes can also initiate a direct attack on pathogens or their propagules, a mechanism commonly known as mycoparasitism (Ownley et al., 2008). Endophytic fungi are known to produce lyase, which effectively aids the evasion of the pathogen and destruction of the pathogen cell walls. This potential mechanism was demonstrated by Grosch et al. (2006) using three different strains of *Trichoderma* sp., which were able to penetrate the hyphae of *R. solani*. Many species of endophytic fungi are known to exhibit predatory behaviors against plant pathogens. The activity is common under nutrient-deficient conditions, and the mechanism is generally termed microbial predation. For instance, Gao et al. (2010) indicated in their report the potential of *Trichoderma* sp. to produce an array of enzymes known to attack the cell walls of fungal pathogens. Similarly, *B. bassiana* has the capability of improving plant growth and causing a reduction in disease severity, even in the presence of plant pathogens. The mechanisms for causing a reduction in the activities of the pathogens were related to competition for space and parasitism and ISR in the *B. bassiana*-colonized plants (Ownley et al., 2008). Similar observations have been reported for *M. anisopliae* and *B. bassiana* in strawberry plants against *B. cinerea* and *Rhizopus* spp. (Dara, 2019a). Both fungi also offered protection for strawberry plants against *Macrophomina phaseolina* (Tassi) Goid, the causal organism of seedling blight (Dara et al., 2018), and treated corn plants against *Fusarium graminearum* Schwabe (Rivas-Franco et al., 2019).

## Plant-Endophytic Fungi Symbiosis and Host Growth Promotion

The findings of the various greenhouse and field trials on fungal endophytes have revealed multiple additional roles of EPF, besides the well-publicized roles as insect killers and plants pathogen antagonists (Vega et al., 2009; Vega, 2018). In addition to the ability of fungal endophytes to induce systemic resistance against herbivores and pathogens, other benefits, such as enhancing drought resistance, inducing tolerance to heavy metals, improving plant fitness under environmental extremes, and promoting general plant growth (biofertilizers), have been mentioned (Tadych et al., 2009; Vega et al., 2009).

Many previous studies and some new publications have provided evidence of the ability of endophytic EPF to promote plant growth, either when existing naturally or when artificially introduced into host plants using various kinds of artificial inoculation techniques (Kabaluk and Ericsson, 2007; Elena et al., 2011; Sasan and Bidochka, 2012; Liao et al., 2014; Lopez and Sword, 2015; Jaber and Enkerli, 2016, 2017; Dash et al., 2018; Bamisile et al., 2020). The capacity of fungal endophytes to colonize plant tissues, establishing a strong symbiotic association with their hosts, has now been well established by various researchers (Kumar et al., 2017). This beneficial association between the two organisms results in plant growth enhancement and improvement of the host’s ability to withstand abiotic and biotic stressors (Saravanakumar and Samiyappan, 2007).

Fungal endophytes are now commonly applied for crop and yield improvement, as they are generally considered eco-friendly, affordable, and renewable sources of nutrients to plants (Kumar et al., 2017). In addition, endophytic fungi also serve as a close alternative to chemical fertilizers when acting as biofertilizers, thereby reducing the heavy dependence on these synthetic compounds (Pal et al., 2015). Many species of endophytic EPF, including *M. anisopliae*, *M. brunneum*, *M. robertsii*, *B. bassiana*, *Purpureocillium lilacinum*, and several others, have been acknowledged for their plant growth promotion potential (Jaber and Enkerli, 2017; Bamisile et al., 2018a,b; Jaber, 2018). The ability to improve plant nutrient uptake, root hair density, and dry weight has been reported for *M. anisopliae*. This is evident in the improved growth and enhancement of root hair density recorded in switch grass and common beans (Sasan and Bidochka, 2012; Behie et al., 2015) and the increase in plant dry weight (biomass) recorded in mung bean, *Vigna radiata* (Rekadwad et al., 2016) following artificial inoculation of the plants with a conidial suspension of the fungus. In addition, the fungus also improved the plant height, root length, root and shoot dry weight of treated tomato seedlings (Elena et al., 2011; Qayyum et al., 2015), foliar biomass, leaf collar formation, and total yield in corn plants (Kabaluk and Ericsson, 2007; Liao et al., 2014) and significantly promoted root development in peanuts (Liu et al., 2017b). Similarly, another fungal species belonging to the genus *Metarhizium*, *M. robertsii*, has also been shown to improve growth in switch grass, corn, wheat, sorghum, tomato, and common beans (Reddy et al., 2009; Elena et al., 2011; Sasan and Bidochka, 2012; Liao et al., 2014). *Metarhizium brunneum* has also been reported as a plant biomass and yield promoter and was also found to improve the nitrogen and phosphate contents, as well as the efficiency of water utilization in colonized plants (Dara, 2019b). This important property of endophytic fungi has also been demonstrated for *B. bassiana*, *B. brongniartii*, *L. lecanii*, *I. fumosorosea*, and several other endophytic insect-pathogenic fungal species (Lopez and Sword, 2015; Jaber and Enkerli, 2017; Dash et al., 2018; Bamisile et al., 2019).

In addition to growth and plant yield promotion, the mutual interaction between the plants and their fungal colonizers also initiates protection for the hosts against unfavorable environmental conditions, such as drought, frost, and heavy metals (Gao et al., 2010). The host defense mechanism against phytopathogenic microorganisms is also enhanced *via* this same interaction. The overall increase in plant growth could also mediate vigor enhancement and resistance to various kinds of biotic and abiotic stressors (Kuldau and Bacon, 2008).

## Mechanisms by Which Endophytic Fungi Act as Plant Growth Promoters/Biofertilizers

Fungal endophytes promote plant growth and host resistance to environmental stressors using various modes of action (Yadav, 2018). The mode of action utilized by these endophytic fungi could be in the form of direct or indirect mechanisms. The ability of fungal endophytes to improve plant growth due to acquisition of nutrients or production of growth-promoting phytohormones is considered a direct mechanism (Hiruma et al., 2018). Fungal endophytes directly improve the rate of growth and development of their hosts by secreting plant growth-promoting hormones, which in turn contributes to improvement of host nutrition using bidirectional transfer of nutrients. The health status of host plants is also improved by protection against phytopathogens (Shen et al., 2019). The rate of phytohormone synthesis mediated by these fungal endophytes varies from plant to plant, with a significant level of effect on the growth, development, morphology, and structure of the hosts (Bamisile et al., 2018a). Concerning plant growth promotion, endophytic fungi are believed to utilize similar mechanisms as rhizobacteria. Several bioactive compounds have been identified to be closely linked to growth promotion in endophytically colonized plants, including auxins (Dutta et al., 2014), gibberellic acid (Khan et al., 2014), cytokinins, and ethylene (Kang et al., 2012). Indole acetic acid (IAA) and the rest of these biocompounds regulate plant physiology, including plant cell division, differentiation and extension, root and xylem development, seed and tuber germination, overall vegetative growth, metabolite biosynthesis, and formation of pigments and photosynthesis (Gao et al., 2010). The insect-pathogenic fungus, *M. robertsii*, has been demonstrated to promote *Arabidopsis* seedling growth (Liao et al., 2017), where the fungus boosted lateral root growth and root hair development using what the authors described as an auxin (IAA)-dependent mechanism. In addition, the fungus activated IAA-regulated gene expression in IAA-deficient mutants and consequently reduced the root hair defects in the mutants. Other strains belonging to *Metarhizium* sp. and *Beauveria* sp. were also found to synthetize auxins (Liao et al., 2017).

Another growth-promoting mechanism is the ability of endophytic fungi to increase the rate of nutrient transport genes in their colonized hosts. This was demonstrated in the study of Behie et al. (2012), where the insect-pathogenic fungus *M. robertsii* was found to transfer nitrogen from the larvae of *Galleria mellonella* Linnaeus to the plant. Similar findings were also reported by Behie and Bidochka (2014), where this same fungus, *M. robertsii*, together with other strains of *B. bassiana*, *M. brunneum*, and *M. guizhouense*, improved the productivity of soybean, wheat, green bean, and switch grass plants through the transfer of insect-derived nitrogen to the host plants. The overall improvement in plant growth due to plant-fungal symbiosis was evident in the ability of the plant to supply photosynthates to the fungi in exchange for the provided insect-derived nitrogen, as demonstrated by Behie et al. (2017).

Many species of plant root colonizing insect-pathogenic fungi, due to their microbial activities, can change the bioavailability of many soil nutrients, hence making them readily available for plant use. This ability involves the mineralization of elements such as nitrogen, iron, potassium, and phosphorus. The latter element, which is the second most essential nutrient (after nitrogen) for plant growth, could be converted from insoluble phosphate into soluble forms and made readily available for plant uptake, a process known as phosphate solubilization (Adhikari and Pandey, 2019; Tandon et al., 2020). Several species of EPF have now been implicated in the production of different forms of organic acids and siderophores, which are small molecular compounds that are known for their ability to make iron readily available for plants (Yadav, 2018).

To this end, several other available reports on endophytic fungus-plant interactions have indicated that plant growth promotion is generally due to the fixation of nutrients, bioactive metabolite production, and synthesis of plant growth-promoting hormones/phytohormones in colonized plants (Behie et al., 2012, 2017).

## Additional Beneficial Applications of Endophytic Fungi

Fungal endophytes play significant roles in IPM programs and have been found to be able to influence plant activities in many ways. As we have discussed in the previous sections, research into endophytes is attracting more interest due to their roles in biocontrol, plant growth promotion, and their potential application in the near future as a replacement/alternative to chemical pesticides and inorganic fertilizers (Shen et al., 2019). However, aside from the aforementioned activities, there are still many more roles and attributes that have been linked to these essential endophytic microorganisms. In fact, many authors have put forward suggestions that the role of fungal endophytes in plant fitness is far from completely defined and should be fully investigated (Vidal and Jaber, 2015; Vega, 2018; Fadiji and Babalola, 2020b).

## Environmentally Safe Alternatives to Chemical Pesticides

The safety of users, other humans, animals, plants, natural enemies, pollinators, and the general ecosystem are the major public concerns related to the application of EPF for the biological control of pests and phytopathogens. Several studies have been conducted in line with ecotoxicological assessments of various EPF (Siegel and Shadduck, 1990; Goettel and Jaronski, 1997; Vestergaard et al., 2003). Studies were conducted to examine the potential side effects resulting from the application of fungal entomopathogens. Roberts (1977) conducted the first study on the detrimental effects of *M. anisopliae* on fish. Following conidia application to waterbodies, the author found no significant effects on the mortality of the examined fishes. Similar observations were recorded in studies conducted to examine the negative effects of *M. anisopliae* on the northern leopard frog, *Rana pipiens* Schreber, and African clawed frog, *Xenopus laevis* Daudin (Peveling and Demba, 2003). Another strain of *M. anisopliae* var. *acridum* formulated into a mycoinsecticide commercially known as green muscle for desert locust control was also tested against the fringe-toed lizard, *Acanthodactylus dumerili* Milne-Edwards. However, no negative effect of the fungus was recorded on the treated lizards following inhalation of conidia, oral exposure, and feeding with mycosed *S. gregaria* locusts. In contrast, *A. dumerili* was found to be greatly affected by the synthetic insecticide fipronil (Peveling and Demba, 2003).

Similarly, toxicity assessments of different strains of EPF have been carried out on several bird species, where birds were reared on EPF-infected insects or directly fed with fungal spores deposited in their feeds. For instance, the ring-necked pheasants, *Phasianus colchicus* Linnaeus were exposed to *B. bassiana* conidia (Johnson et al., 2002), chickens were fed *B. brongniartii*-infected white grubs, and American sparrowhawks, *Falco sparverius* Linnaeus were equally fed spores of *B. bassiana* (Althouse et al., 1997). In the aforementioned studies, histopathological changes were not reported in any of the treated birds, whereas no significant differences were reported among the control and treated samples with regard to the growth, body mass, and survival of birds (Zimmermann, 2007a).

Toxicity tests on *B. bassiana* conducted on rats and other vertebrates also confirmed the non-toxicity of the fungus (Goettel and Jaronski, 1997). Intramuscular injection of *B. bassiana* into mice indicated that the fungus could only survive for 3days inside the rodents (Semalulu et al., 1992).

Another study that was conducted by Zimmermann (1992) to investigate the vertical movement of wet and dry spores of *M. anisopliae* also confirmed that the possibility of contamination of the groundwater by the fungus is very low. When insect-pathogenic fungi and all kinds of mycopesticides are applied for biocontrol, the water bodies and the atmosphere are arguably the clear destinations for drifting formulations. However, according to Weng et al. (2019), until now, there has been no available record of the negative effects of EPF from water and the atmosphere on human health. The reason for this is the inability of fungal spores to persist or proliferate in the atmosphere for a long duration (Shah and Pell, 2003). Milner et al. (2002) also concluded that the possibility of *Metarhizium*-based biopesticides posing negative effects on aquatic living organisms is relatively low. Most mycotoxins that are commonly known as environmental or food chain pollutants have now been found to be produced as a result of plant infection by fungal phytopathogens, rather than endophytic colonization of the plant by the fungal entomopathogens (Oyedele et al., 2017; Weng et al., 2019). Common examples are the mycotoxins produced by *Fusarium* spp., *Aspergillus* spp., and other fungal phytopathogens, which have been found to contaminate the environment through the crops and products they infect (Oyedele et al., 2017; Mallebrera et al., 2018).

Several recent studies have also presented reports on the ability of endophytic fungi to initiate the production of phytohormones and other bioactive compounds related to plant growth promotion, thereby improving the overall development and growth of colonized host plants. As a result, we can now anticipate a potential decline in the level of dependence on synthetic fertilizers, which are notorious for ecosystem pollution due to their residual effects and tendencies to enter the food chain. It is also noteworthy that during the development and registration of any EPF for use as a mycopesticide, the fungus is extensively examined for safety against many beneficial non-target organisms. In this light, there is an overall tendency of biological control agents to be safer for use than chemical products. There is therefore a strong need to create awareness and inform policy and regulatory authorities on the safety and advantages of using biopesticides compared to their synthetic counterparts.

## Compatibility with Other Biocontrol Agents

The compatibility of several species of EPF with many other biological control agents, especially the associated natural enemies of targeted pests, such as predators (Canassa et al., 2019) and parasitoids (Akutse et al., 2014; Gathage et al., 2016; Jaber and Araj, 2018), has widely been reported. For instance, the potential management of green peach aphids, *Myzus persicae* Sulzer (Hemiptera: Aphididae) in sweet pepper using the parasitoids of green peach aphids, *Aphidius colemani* Viereck (Hymenoptera: Braconidae) in combination with *B. bassiana* and *M. brunneum* was demonstrated by Jaber and Araj (2018). Similarly, according to Akutse et al. (2014), the pea leafminer, *Liriomyza huidobrensis* Blanchard (Diptera: Agromyzidae) can be controlled using two parasitoid species, *Diglyphus isaea* Walker (Hymenoptera: Eulophidae) and *Phaedrotoma scabriventris* Nixon (Hymenoptera: Braconidae), in combination with different fungal isolates, including *B. bassiana* and *H. lixii*. The potential utilization of two isolates of *B. bassiana* and *M. robertsii* in combination with the predatory mite, *Phytoseiulus persimilis* Athias-Henrio (Acarina: Phytoseiidae) for the management of the two spotted spider mites, *Tetranychus urticae* Koch (Acari: Tetranychidae) on strawberry plants in the greenhouse (Canassa et al., 2019) and in the strawberry field (Canassa et al., 2020) has also been demonstrated. Similarly, studies have revealed the possibility of applying *L. lecanii* in combination with an aphid alarm pheromone and sublethal doses of the insecticide imidacloprid as part of an autodissemination strategy to enhance the efficacy of the fungus for aphid biocontrol (Hartfield et al., 2001). Another typical generalist insect-pathogenic fungal species that has been tested for compatibility with other biocontrol agents is *Zoophthora radicans* Brefeld (Zygomycetes: Entomophthorales). The fungus was applied in an autodissemination technique for the management of the diamondback moth, *Plutella xylostella* Linnaeus (Lepidoptera: Yponomeutidae) in combination with semiochemicals. The host-specific semiochemical attracted the insects into an inoculation device, where the moths were exposed to the conidia of *Z. radicans* (Pell et al., 1993). The combined use of *B. bassiana*, *M. anisopliae* and different chemical fungicides in harvested strawberry was also to be found suitable for the management of *B. cinerea* and *Rhizopus* sp. (Dara, 2019a).

Most biocontrol approaches benefit from being used together, therefore, to improve the efficacy of specific biological control approaches, it is imperative to apply them in an integrative manner in combination with other cultural or conventional measures, as the synergy would benefit both biocontrol agents and significantly suppress pest populations. In addition, classical and inoculation methods could also be applied in combination with conservative methods in the quest to increase the efficiency of both approaches (Pell et al., 2001).

## Endophytic Fungi as Good Sources of Pharmaceutical Products

Endophytic fungi are not just biocontrol and plant growth-promoting agents, but they have now been established as good sources of various arrays of medicinal or pharmaceutical products. The endophytic microbes constitute an important source for drug discovery, and their plant sources are being extensively explored for new chemical compounds for therapeutic purposes (Tadych et al., 2009; Fadiji and Babalola, 2020a). Fungal endophytes act as reservoirs of novel bioactive secondary metabolites, such as alkaloids, phenolic acids, quinones, steroids, saponins, tannins, and terpenoids, that serve as potential candidates with antimicrobial, anti-insect, anticancer and many more properties (Gouda et al., 2016). A variety of products derived from bioactive secondary metabolites belonging to different endophytic fungal species has now been developed for use as antibiotic agents such as cephalosporin and penicillin (Tadych et al., 2009). These biocompounds have also been explored for their antimalarial, antiarthritis, anticancer, antidiabetic, antiviral, antituberculosis, anti-inflammatory, and immunosuppressive potentials (Tadych et al., 2009; Fadiji and Babalola, 2020a). The products are isolated and utilized in their raw forms or otherwise formulated to produce different drugs for the treatment of many health conditions (Hu et al., 2016).

Data collected over the last four decades have listed over 70 novel secondary metabolites derived from *Isaria* sp. alone (Weng et al., 2019). For instance, a non-ribosomal peptide metabolite known as beauvericin, which is a cyclic hexadepsipeptide mycotoxin isolated from a strain of *I. fumosorosea*, has been found to possess insecticidal, antibacterial, antiviral, and cytotoxic properties and is considered valuable for the formulation of new pesticides (Lu et al., 2016). Additionally, fumosorinone, a terpene compound isolated from the same fungal species, could act as a classic non-competitive inhibitor of protein tyrosine phosphatase 1B (PTP1B), indicating that the compound could function in medicine for the clinical treatment of diabetes (type II) and other related metabolic disorders (Liu et al., 2015). This same compound has also been related to cytotoxicity against human cancer lines (Chen et al., 2018). Peroxyergosterol is a biocompound isolated from another strain of *I. fumosorosea* and has been tested for various bioactivities, including its cytotoxicity against cancer cells (Sheu et al., 2000), apoptosis of human leukemia cells (Takei et al., 2005), and potential production of vitamin A (Zhang et al., 2013).

In addition, there are several reports of the isolation and identification of many other biochemical compounds of medicinal importance from other fungal species, including *B. bassiana*, *B. brongniartii*, and *M. anisopliae* (Zimmermann, 2007a,b). To this end, endophytic microbes are now commonly utilized in the mass production of drugs, enzymes, antibodies, supplements, and riboflavin, among many other industrial products (Latz et al., 2018). Isolated microorganisms are of huge importance in the fields of medicine, agriculture, and industry (Sahay et al., 2017).

## Entomopathogenic Fungi as Alternatives to Chemical Pesticides: What Are the Challenges?

The huge importance of insect-pathogenic endophytic fungi and their derived biocompounds to agriculture, industry, and medicine cannot be overemphasized. However, despite their numerous attributes and functions, several problems affecting their successful application as biological control agents have been identified. One of the major challenges is the difficulties in isolation and identification of fungal endophytes. As many fungal endophyte strains have been found to be unculturable, measuring and identifying the endophyte community structure and diversity has been a difficult task (Fadiji and Babalola, 2020b). Even though, very recently, scientists in advanced countries have found alternative ways to isolate and identify novel fungal strains, especially by employing various cultivation-independent techniques. However, there is every possibility that a larger percentage of fungal isolation and identification efforts still depend heavily on traditional culture methods with selective media. The adverse effects of geographical location, vegetation type, and human disturbance on fungal entomopathogen distribution are another problem. The irregular localization or biodiversity of fungal entomopathogens in soils as a result of geographic and climatic conditions has been reported as a major disadvantage. For instance, in a study conducted across the Qinghai-Tibet Plateau and Gansu Corridor of China in 2016, it was reported that the likelihood of isolating novel strains of fungal entomopathogens is higher in areas characterized as remote and less disturbed by human activities (Dong et al., 2016). The soil types, vegetation or landscapes, habitat fragmentation and alteration, and climatic conditions are some of the determining factors that are also related to endophytic fungal richness and diversity in the soil. The negative influence of environmental conditions, such as temperature, humidity, and solar radiation, on fungal entomopathogen virulence and persistence in the field has also been investigated (Zimmermann, 2007b). Another limiting factor is the rapid decline in the level of efficacy of EPF over a short duration. As a result, fungal-based mycopesticides are generally not highly regarded as alternatives to chemical pesticides among users. The possibility of posing unwanted residual effects on predators, parasitoids, pollinators, and other non-target organisms has also been mentioned. This activity has been examined in some insect-pathogenic fungal species with a broad spectrum and wide host range, such as *B. bassiana* and *B. brongniartii* (Goettel et al., 1990). Some previous studies investigated various possible adverse effects on beneficial insects, earthworms, honeybees, vertebrates, and plants (Vestergaard et al., 2003). Although most of the studies were conducted in the laboratory and only a few were field trials, many of these studies argued that EPF could be used with little or no side effects on non-target organisms (Goettel et al., 1990; Vestergaard et al., 2003). Nevertheless, as with every general principle, there could be some exceptions across species and perhaps even among isolates. There are possibilities that different isolates within the same species can perform very differently even on the same host. For instance, insect host range, fungal infection levels, rate of germination, and temperature optima can vary among fungal species and isolates (Zimmermann, 2007a). To increase the adoption of these mycopesticides, it is also important to develop a “new paradigm” for applying these entomopathogens as opposed to the “old paradigm” of application in ways similar to their synthetic chemical counterparts.

## Overview of the Research Advances in the Last Few Decades and Insights for Next-Generation Sustainable Agriculture

Several of the previous studies on fungal endophytes and other related studies have focused on co-culturing methods in an *in vitro* dual plate assay examine the antagonistic effects of endophytic fungi against some targeted pathogens. Many of the highlighted studies only indicated endophytic fungal antagonistic effects on the target plant pathogens without necessarily conducting comprehensive assessments of the physiological changes in the colonized plants. Another strategy that is commonly adopted is to compare the treated and untreated seedlings following artificial inoculation of plants with pathogens with respect to the rate of survival, colonization rate, and disease severity index (Jaber, 2018). The mechanisms by which endophytic fungi mediate changes in host physiology and volatile levels also have yet to be fully explored. The available data are limited and have shown inconsistencies under various environmental conditions (Fontana et al., 2009). However, in addition to the aforementioned descriptive studies, in the last few decades, a good number of emerging studies have been conducted to further explore the ecology of fungal endophyte-plant host specificity and their multitrophic effects (Hartley and Gange, 2009). During that period, the molecular mechanisms related to fungal endophyte-induced host plant defense were an area of increasing focus and research interest (Zheng and Dicke, 2008).

Since the beginning of the biotechnology revolution, scientific research has been focused on genetic engineering of fungal endophytes with the sole aim of improving plant yields and their defense systems (Clay, 1994). With the introduction of gene modification procedures in EPF (Wang and St Leger, 2007) and the progress of RNAi technology, studies are now targeted at constructing recombinant fungal strains with enhanced virulence (Chen et al., 2015). Genetic engineering could therefore provide useful strategies to increase fungal virulence or enhance fungal resistance to different stress factors. Over time, the utilization of recombinant endophytic genes as biocontrol agents has become popular and of huge importance. Recombinant endophytic organisms produce anti-pest proteins for insect pest management, and they can also successfully colonize host plants (Fadiji and Babalola, 2020b). However, it is worth noting that recombinant endophytic fungi with enhanced virulence against insects may represent a risk for pollinators and beneficial insects (natural enemies). In the efforts to understand the chemical pathways that are applicable in biotechnological applications, the transfer of genes from associated endophytic fungi to the genome of their hosts toward the production of secondary metabolites has been one of the principles used for explaining the multiple origins of chemical defenses within the phylogeny of different plant species (Wink, 2008). In recent times, advances in microbial biotechnology have translated into the biotransformation of many chemicals in the quest of reducing environmental pollution. Novel techniques such as bioremediation, waste management, and composting represent forms of technological advancement from the crude method of metabolite synthesis involving only ethanol and butanol. In recent times, various scientists have focused on exploring the world of microorganisms, plants, and animals for their potential utilization in the production of novel medicinal products (Gouda et al., 2016; Latz et al., 2018). It is now evident that products derived from natural sources are less expensive and user- and ecosystem-friendly (Fadiji and Babalola, 2020a).

The integrated use of EPF, such as *B. bassiana*, in combination with other chemical pesticides has been investigated. There are suggestions that the adoption of the combination would help improve resistance management strategies and reduce ecosystem pollution due to excessive use of inorganic insecticides (Al-Ani et al., 2021). In the past few decades, the combined application of biological control agents through an autodissemination strategy has also recorded a level of success. For EPF in particular, this strategy has proven successful for many strains when using EPF in combination with semiochemicals and other insect natural enemies (Vega et al., 2000).

Over the years, examination of plant-endophyte symbiosis has gone beyond culture media assays, as there are pressing needs to analyze many other non-culturable endophytic fungal species using culture-independent methods (Adeleke and Babalola, 2021). In this light, more comprehensive methods, such as microscopic observation of fluorescent mycelia and confocal scanning electron micrographs enabled by green fluorescent protein (GFP) labeling, have recently been employed (Sasan and Bidochka, 2012; Behie et al., 2015). The latter approach would enable observation of intercellular and intracellular endophytic localization of the endophytic fungi in the treated plants (Behie et al., 2015).

To gain more insights into the molecular mechanisms associated with plant responses to endophytic fungal colonization, metagenomic analysis of different plant organs for prospective fungal colonizers can be conducted. The analysis would help to examine the functions, structures, and phylogenetic construction of genetic relatedness in the microbial genomes from long reads of metagenome sequence data (Adeleke and Babalola, 2021). For studies related to genome structure and features, molecular techniques that are now commonly used include polymerase chain reaction (PCR), DNA sequencing, DNA microarrays, and RT-PCR (Chen et al., 2015; Wang et al., 2020), among many others. For studies related to endophyte-induced secondary compounds, phytohormones, and enzymes, several molecular analyses are commonly being used. The most common technologies used include liquid chromatography-mass spectrometry (LC-MS-MS; Wang et al., 2020), gas chromatography-mass spectrometry (GC-MS/MS; Elbanhawy et al., 2019), high pressure liquid chromatography-tandem mass spectrometry (HPLC-MS/MS), ultra-performance liquid chromatography-tandem mass spectrometry (UPLC-MS/MS; Cotes et al., 2020), nuclear magnetic resonance spectroscopy (NMR), headspace solid-phase microextraction (HS-SPME), Fourier transform infrared spectroscopy (FTIR), and proteomics (Al-Ani et al., 2021).

Similarly, different types of high-throughput equipment are currently used for fungal DNA sequencing, notably next-generation sequencing methods such as 454 pyrosequencing and Illumina sequencing. This method can enhance the understanding of fungal microbiomes. The latest advances in microbiology research ensure that the discovery, isolation, and identification of novel genetic traits are easier while providing greater insights into the underlying mechanisms of plant-microbe interactions. All microbial communities could be examined from the internal tissues of the plants, with a special focus on the novel genes responsible for host growth improvement, phytohormone synthesis, cellular metabolism, and nitrogen fixation (Hardoim et al., 2015). Scientists, through metagenomics analysis of the internal tissues of plants, can now detect the specific genes related to plant growth promotion and other physiological functions (Igiehon and Babalola, 2017). Additionally, through knowledge gained with metagenomics, the various studies related to fungal endophytes and other microorganisms are made simpler and more accurate. The application of omics technology has advanced studies on plant-microbe interactions to the level of genomics, proteomics, and transcriptomics (Akinola and Babalola, 2020). With the enormous research progress and technological advancements made in the past couple of decades, endophytic fungi and their bioactive compounds are arguably suitable for adoption as replacements of inorganic fertilizers and chemical pesticides if carefully explored by researchers and embraced by policymakers (Fadiji and Babalola, 2020b).

## Conclusion

The latest advances in microbiological research have helped to establish the importance of microorganisms in the fields of medicine, industry, and agriculture. An in-depth understanding of the roles of these beneficial microorganisms will enhance the exploitation of their ecosystem services and their successful adoption and optimum utilization in agriculture, especially as plant growth- and crop yield-promoting agents. Concerns about the negative effects of synthetic chemical pesticides have also driven attention toward developing eco-friendly pest management techniques. The various species of insect-pathogenic fungi, fungal endophytes, and other beneficial microorganisms that could function as biocontrol agents are now generally considered sustainable pest management options for incorporation into IPM programs or as a substitute/supplement for chemical pesticides. Overall, the potential applications of mycopesticides as alternatives to chemical pesticides are promising; however, there is still much work to be done to fully explore their services. Based on the available pieces of evidence, most EPF-based pesticides are considered to be relatively safe for use and could effectively mitigate the abuse of synthetic pesticides. Nevertheless, with respect to future registrations of new fungal strains, it is imperative to conduct pathogenicity/toxicity-related tests in non-target organisms, as well as for vertebrates, to avoid potential risks. It has, however, been suggested that all risks cannot be excluded; nevertheless, efforts should be put in place to ensure that existing precautionary measures during production and application are taken to avoid harmful reactions. There are no specific criteria that guarantee the acceptance or adoption of fungal biocontrol agents, but efforts are warranted to promote the use of bioproducts from these microorganisms due to their numerous advantages. The various underlying problems that need to be solved will not only be addressed by laboratory or field trials but also at the policy and regulatory levels. In addition to scientific aspects, economic, social, and political limitations must also be addressed to fully explore the potential uses of these microorganisms.

## Author Contributions

BB and KA designed the review outline. The manuscript was written by BB and JS. KA and YX reviewed the manuscript. All authors have read and agreed to the final version of the manuscript.

## Funding

We acknowledge funding support received through Laboratory of Lingnan Modern Agriculture Project (NZ2021022) and the UK’s Foreign, Commonwealth & Development Office (FCDO; B2329ADFID-FAW and B2291A-FCDO-BIOPESTICIDE) through the International Centre of Insect Physiology and Ecology (*icipe*).

## Conflict of Interest

The authors declare that the research was conducted in the absence of any commercial or financial relationships that could be construed as a potential conflict of interest.

## Publisher’s Note

All claims expressed in this article are solely those of the authors and do not necessarily represent those of their affiliated organizations, or those of the publisher, the editors and the reviewers. Any product that may be evaluated in this article, or claim that may be made by its manufacturer, is not guaranteed or endorsed by the publisher.
