# Supplementary figures and images for: Model Application of Entomopathogenic Fungi as Alternatives to Chemical Pesticides: Prospects, Challenges, and Insights for Next-Generation Sustainable Agriculture
Source: Front Plant Sci. 2021 Sep 30;12:741804. doi: 10.3389/fpls.2021.741804 (PMC8514871; doi:10.3389/fpls.2021.741804)

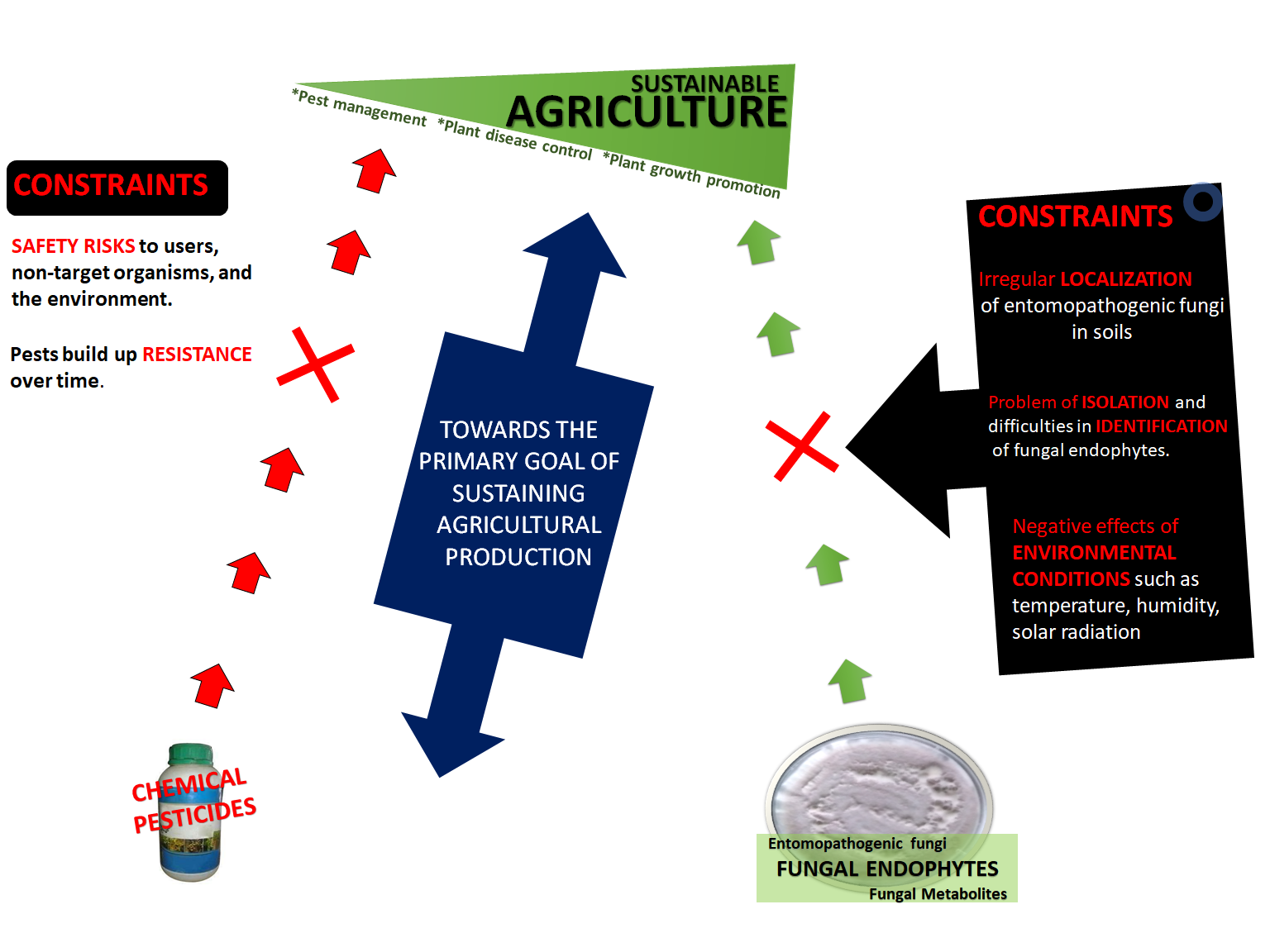

Supplement: Supplementary file 1 [file Image_1.PNG]
